# Do Alarmins Have a Potential Role in Autism Spectrum Disorders Pathogenesis and Progression?

**DOI:** 10.3390/biom9010002

**Published:** 2018-12-20

**Authors:** Eleonora Di Salvo, Marco Casciaro, Sebastiano Quartuccio, Lucrezia Genovese, Sebastiano Gangemi

**Affiliations:** 1 National Research Council of Italy (CNR), Institute of Biological Resources and Marine Biotechnologies (IRBIM), 98122 Messina, Italy; lucrezia.genovese@iamc.cnr.it; 2 National Research Council of Italy (CNR), Institute of Applied Science and Intelligent System (ISASI), 98164 Messina, Italy; 3 School and Operative Unit of Allergy and Clinical Immunology, Department of Clinical and Experimental Medicine, University of Messina, 98125 Messina, Italy; mcasciaro@unime.it (M.C.); gangemis@unime.it (S.G.); 4 Mezzogiorno d’Italia “Franco Scalabrino” Orthopedic Istitute, 98165 Messina, Italy; sebastiano.quartuccio@yahoo.it

**Keywords:** autism, alarmins, autism spectrum disorder, S100, HMGB-1, HSP, IL-33, immune system, inflammation

## Abstract

Autism spectrum disorders (ASDs) represent a disabling condition in early childhood. A number of risk factors were proposed in order to explain their pathogenesis. A multifactorial model was proposed, and data supported the implication of genetic and environmental factors. One of the most accepted speculations is the existence of an imbalance of the immune system. Altered levels of cytokines, chemokines and immunoglobulins were demonstrated in patients with ASDs; in particular, proinflammatory mediators were significantly increased. Alarmins are a multifunctional heterogeneous group of proteins, structurally belonging to specific cells or incorporated by them. They are released in the surrounding tissues as a consequence of cell damage or inflammation. Their functions are multiple as they could activate innate immunity or recruit and activate antigen-presenting cells stimulating an adaptive response. Alarmins are interesting both for understanding the inflammatory process and for diagnostic purposes as biomarkers. Moreover, recent studies, separately, showed that alarmins like interleukin (IL)-33, high-mobility group box 1 (HMGB1), heat-shock protein (HSP) and S100 protein (S100) could play a relevant role in the pathogenesis of ASDs. According to the literature, some of these alarmins could be suitable as biomarkers of inflammation in ASD. Other alarmins, by interfering with the immune system blocking pro-inflammatory mediators, could be the key for ameliorating symptoms and behaviours in autistic disorders.

## 1. Background

Leo Kanner and Hans Asperger elaborated the expression “Infantile Autism” for the first time in the 1940s. According to their definition, the subjects observed had a disturbance of personality and of cognitive perceptions [1,2]. The cases described aroused a lot of interest and today these affections are known as “autism spectrum disorder” (ASD). The list of disorders includes a group of different brain-based neuro-developmental disorders with growing grades of symptom severity. ASD represents a disabling condition in early childhood; a number of risk factors were proposed in order to explain its pathogenesis, without reaching a unique hypothesis [3]. The average age of diagnosis is at approximately four years of age and is a lifelong disorder. A multifactorial pathogenesis has been proposed; several pieces of evidence supported the implication of genetic and environmental factors [4].

For sure, it emerged that a dysfunction of the central nervous system, especially during the neurodevelopment might provoke the genesis of behavioural and cognitive disorders. Epigenetics is defined as the regulation of gene expression by producing functional changes in DNA without altering the genomic sequence. It demonstrated being a fundamental component for brain development, aging, and many central nervous system (CNS) disorders. In fact, epigenetics plays a main role in the plasticity phases of brain development and so it is important for the aetiology of CNS disorders [5]. Epigenetic during the pre-natal brain development is capable of re-programming functional genes associated to a higher risk of psychiatric or neurodegenerative disorders [6,7]. Its role, in a near future, has to be better clarified. However, the changes induced in histone and DNA by the methylation seem to be the most involved processes. A complete understanding of these changes could bring to the development of new therapeutic approaches [8].

Several diseases like gastrointestinal, psychiatric and autoimmune ones were found to have an intimate link with ASDs. In fact, autistic children often have a high prevalence of immune-related pathologies such as allergies and autoimmune diseases [9,10,11,12,13,14,15,16]. According the scientific world, one of the most accepted speculations surrounding autism is an imbalance of the immune system [17]. A study reported that an early activation of innate immunity during pregnancy, mainly due to an infectious disease, could considerably augment the possibility of being affected by autism [18]. Altered levels of cytokines, chemokines and immunoglobulins were demonstrated in patients with ASDs; in particular, proinflammatory mediators were significantly increased (interleukin (IL)-1β, IL-6, tissue growth factor (TGF), interferon-γ and nuclear factor kappa-light-chain-enhancer of activated B cells (NF-kB)) [16].

Alarmins are a multifunctional heterogeneous group of proteins, structurally belonging to specific cells or incorporated by them. They are released in the surrounding tissues as a consequence of cells damage or inflammation. Their functions are multiples as they could activate innate immunity or recruit and activate antigen-presenting cells (APC) stimulating an adaptive response [19].

Their main function is to ameliorate the immune system response. On the other hand, they could compromise the homeostasis by leading to a massive pro-inflammatory response. This last effect could constitute the basis for many diseases [20].

According to these pieces of evidence, alarmins are interesting both for understanding inflammatory processes and for diagnostic purposes as biomarkers. Moreover, recent studies, separately, showed that alarmins like IL-33, high-mobility group box 1 (HMGB1), heat-shock protein (HSP) and S100 protein (S100) could play a key role in the pathogenesis of ASD. For this reason, the purpose of this review is to examine and to understand the possible link between ASD and the family of alarmins and to clarify the involvement of the immune system. A list of studies about alarmins in ASD are displayed in Table 1.

## 2. Interleukin-33

Interleukin-33, identified as an “alarmin, belongs to the IL-1 family cytokine and acts intracellularly as a nuclear factor regulating gene expression. It exerts its function through the suppressor of tumorigenicity 2 (ST2) receptor, which is highly and specifically expressed on Th2 cells [35]. Two isoforms of this receptor were identified: a trans-membrane form T1/ST2, and soluble one sST2 [36]. Interleukin-33 is implicated in many diseases; especially those were immune system imbalance is fundamental [37].

The recently discovered innate type 2 lymphoid cells (IL-C2s) constitute an immune cell population expressing the ST2 receptor; they are produced in response to an IL-33 cellular increase. Innate type 2 lymphoid cells are able to secrete several cytokines such as IL-4, IL-5, IL-9 and IL-13. As a consequence, they are involved in type 2 immune response, allergies, and parasitic infections [38,39,40].

Recently, the involvement of immune system cells became prominent. Some authors individuated the ability of IL-33 to amplify the proinflammatory response of ILC2 cells. In fact, these immune cells after the stimulation by IL-33 produce IL-4, IL-5, IL-9, IL-13 [41]. Also, mast cells are able to generate such cytokines giving the start to a type-2 inflammation. The above-cited mediators resulted being elevated in the serum of pregnant women giving birth to autistic children and in the amniotic fluid of future autistic patients [42]. Moreover, autistic subjects had a positive association with allergic diseases, such as atopic dermatitis and asthma, confirming the immune pattern triggered by IL-33, ILC2 and mast cells [43,44].

The role of IL-33 in ASDs is still unknown, but a meta-analysis showed an increase in the levels of pro-inflammatory cytokines and a reduction in the concentration of the anti-inflammatory cytokines. These results suggest the presence of an acute inflammatory state, supporting the speculation of an immune system dysfunction [45]. A study analysing IL-33 did not found statistical differences between ASD and control subjects. On the other hand, IL-33 levels were positively correlated with sST2 levels [21] and these results were confirmed by other authors [22].

The on-going pro-inflammatory status could be linked to disease severity. The heterogeneity of the results could indicate different immunological pattern associated to ASD severity [46]. An animal study correlated high brain cytokines levels to the disease stage. The higher was the level of IL-33, the more mice showed behavioural disturbances [47]. Saresella et al. demonstrated a significant up-regulation of the AIM2 and NLRP3 inflammasomes and an increased production of IL-1β and IL-18 associated to a reduction of IL-33 in autistic disorders [23].

## 3. High-Mobility Group Box 1

High-mobility group box proteins constitute a family of non-histone and ubiquitous molecules with a pro-inflammatory function. They belong to the high mobility group family of proteins. There are four categories of HMGB, from 1 to 4. High-mobility group box 1 (which is known also as HMGB-1, HMG1, HMG-1, HMG 1, amphoterin, p30) is the most represented of the whole HMG family proteins and is encoded in human by the *HMGB1* gene (13q12) [48,49]. It was demonstrated having a key role in the regulation of the immune system, especially in some diseases such as asthma and chronic obstructive pulmonary disease [50,51].

As demonstrated by Babinska in a recent paper, HMGB1 resulted being significantly increased in ASD samples. Furthermore, this study demonstrated that HMGB1 could play an important role because it promoted neurite outgrowth and cell migration. [4]. In fact, treatment with inhibitors of HMGB1 activity was found being able to reduce the inflammatory response in a wide range of preclinical autism models [52].

Previously, Russo et al. found the same high levels of HMGB1 together with reduced levels of plasma epidermal growth factor, fundamental for the differentiation of cells in the CNS [25,26]. The association of HMGB1 with autism was demonstrated being severity-related. The more it is elevated, the more it worsens the social interaction [24]. The final confirmation of the key role of this alarmin in neuroinflammation was given by the utilisation of HMGB1 inhibitors which considerably reduced the inflammatory response [53]. Although, in these results, there is some risk of bias. In fact, often authors studied ASDs and not only autism, and the studies were performed both on children and adults [52].

## 4. Heat-Shock Proteins

The heat-shock proteins (Hsp) represent a group of molecular chaperones, fundamental for the maintenance of protein homeostasis [54]. In human beings, 332 genes encode for the chaperone and co-chaperone families, and together constitute the “chaperome” [55]. These chaperone genes encode for the heat shock proteins (Hsp), initially identified by their enormous up-regulation during cellular stress conditions. These are divided into several classes, divided according to their monomeric molecular mass, such as Hsp100, Hsp90 and Hsp70 [56].

Several immunological studies suggested autoimmunity as a pathogenic factor in autism.

In particular, serum from children with ASD had higher levels of anti-HSP-60 antibodies. The heat-shock protein-60 is known to be a superantigen, a nonglycosylated low-molecular-weight exoprotein very resistant to high temperatures, which could activate an intense immune response. Together with a positive correlation to anti-gliadin antibodies, these findings supported the idea that in autism an immune system malfunction could have a role [27,57].

Some researchers speculated that the involvement of inflammatory molecules during the pre-natal period could provoke an autoimmune disease causing schizophrenia and an ASD. In fact, pro-inflammatory cytokines and Hsp-90 concentrations resulted in enhancing cellular defences [58]. Cytokine activation axis like NF-kB, JAK/STAT have a main role in CNS cells proliferation and differentiation particularly during pre-natal development [59]. As a consequence, the boost of pro-inflammatory cytokines also caused by Hsp showed the ability to disturb cortical neuron dendrite development causing the outbreak of autism [60].

However, HSPs have a pivotal role in limiting protein misfolding and blocking apoptotic activity; they represent a class of molecules in all probability involved in neurological disorders [61]. In addition, it was demonstrated that a malfunction of Hsp-70 is correlated to oxidative stress, a main actor in ASD’s aetiology [28]. Animal researches proposed that HSP pathways and altered gene expressions concerning innate immunity could contribute to the development of neurological symptoms [62].

## 5. S100 Protein

S100 is a family of highly acidic calcium-binding proteins found in large concentration in the brain and initially believed to be glial. They are also found in other body organs. They have in common the EF-hand motif found on some calcium binding proteins. Initially, the soluble markers for neuron-specific enolase (NSE) and the corresponding glial S100 were found to be suitable in acute phases of brain destruction. Studying its profile in autism, however, Ahlsen et al. did not find any difference between healthy subjects and patients [29].

The S100 family was found having many subcategories, which were studied in neurological disorders. In particular S100A9, an inflammation-associated calgranulin, is highly expressed in a number of inflammatory conditions. S100A9 is a major cytosolic protein in monocytes; the monocyte count is typically elevated in autism. The independent association between S100A9 levels and Childhood Autism Rating Scale (CARS) scores confirmed and expanded previous findings on the role of immune activation in the pathogenesis of ASD [30].

On the other hand, S100B is a calcium-binding protein capable to regulate the cytoskeleton and proliferation of astrocytes. Moreover, S100B acts as a cytokine for neighbouring cells (astrocytes, neurons, and microglia), depending on its levels. This protein, in vitro, at molecular concentration stimulates neurite over-growth and neuronal survival. It is also able to protect hippocampal neurons versus glutamate toxicity. In astrocytes, it would be possible conceive that risperidone stimulates S100B secretion; in turn S100B stimulates neuronal survival and activity during brain injury and recovery. The results demonstrated for the first time that the atypical neuroleptic risperidone can modulate the morphology and S100B secretion in C6 glioma cells. These data contribute to the proposal that glial cells are targeted by risperidone, being involved in the therapeutic response of risperidone in improving autism symptoms [63]. Coffin et al. tested the presence of S100 protein in lipoblastoma bioptic tissue of several patients. Among these was one patient, male, six months old, who was affected by autism which was also immunoreactive for S100 [31]. S100B is produced primarily by astrocytes. An increased serum S100B protein reflects neurological damage. Autoantibodies could pass through the blood–brain barrier and match to brain tissue antigens, creating immune complexes causing neurological destruction. S100B protein levels were elevated in autistic children and significantly correlated to disease severity [32]. Concentrations of both S100B and tumor necrosis factor-alpha (TNF-α) were higher in children [34]. S100B is commonly considered a marker of glial activation in response to brain damage. Additionally, the neurotrophic effect of S100B is not known. In a study performed by Esnafoglu et al., S100B values could not be considered as biomarkers for ASD [33]. Gholizadeh et al. demonstrated the presence of S100β cells in mice brain in a developmental stage suggesting the importance of the protein in a healthy brain [64]. Lee et al. confirmed the importance of S100β in the physiology of a normal brain growth demonstrating its increase as effect of early valproic acid exposure [65].

## 6. Discussion

During the last two decades, researchers of all over the world suggested that immune system could have a predominant role in autism pathogenesis. In particular, autoimmunity was pointed out. In fact, several antibodies against neuron-antigens were dosed. Due to the importance given to the immune response, we collected as much information as possible regarding alarmins role in autism. Very early infections in the infant could lead to the production of proteins capable of modulating immune function. These are the so-called “heat-shock proteins” or super antigens. The induction of such molecules could create an imbalance in the immune system. The work of Vojdani et al. demonstrated the augmentation of HSP-60 in patients affected by ASDs. Heat shock proteins presence together with other autoantibodies supported the idea of an autoimmune pathogenesis of autism [27]. Like HSP, there are other proteins called alarmins, which amplify the inflammatory response. The high mobility group box 1 proteins, belonging to the alarmins group, were pointed as possible causal agents. The high mobility group box 1 proteins in fact, act as powerful proinflammatory cytokines. In most cases their presence is proof of a local damage [4,24,53]. The studies cited above reported how, in autistic children, this alarmin is constantly augmented sustaining the hypothesis of a persistent inflammation in the central nervous system of these patients. The high mobility group box 1 is also constantly augmented in other neurological diseases. As targeting this molecule was experimentally demonstrated being valid in other proinflammatory diseases, following this path also in autism could be a possibility. The neurodevelopment and the neuroarchitecturing are fundamental in autism pathogenesis. Astrocytes together with other glial cells are fundamental in this objective. S100 proteins, belonging to the alarmins family too, were found being expressed at high levels in astrocytes. In particular, S100B are expressed in these glial cells. It was observed that, after a brain injury, S100B levels augmented in the serum of patients, so it was decided to perform researches about S100 concentration in autistic subjects. It emerged that in many cases, S100 levels were elevated [34,63]. In some cases, this augmentation was associated to high proinflammatory cytokines level. Astrocytes are the most abundant source of S100B in human brain; given the importance of these cells in the neurodevelopment and in the neuroarchitecturing, further studies could confirm their role in autism pathogenesis.

Recent and interesting researches showed a close correlation between the intestinal microbiome and ASD. In fact, ASD patients are often affected by gastrointestinal (GI) symptoms such as constipation, bloating and diarrhoea. An altered GI microbiome was found to be present in autistic patients [66,67]. A recent clinical trial conducted by Kang et al. investigated the potential of faecal transplantation in autistic children. Upon completion of the treatment, a significant improvement in gastrointestinal symptoms was observed, including constipation, diarrhoea, indigestion and abdominal pain [68]. According some authors, modifying the microbiota through diet may represent an effective strategy to treat neurological pathology [69]. This intimate connection among microbiome, GI symptoms, behavioural pattern, inflammation, and immuno-modulation could be balanced by an intervention on children homeostasis by targeting alarmins. Some other studies confirmed an important role of alarmins in the gut microbiota. In fact, Malik et al. demonstrated that IL-33 is an important regulator of the microbiome. Specifically, IL-33-deficient mice had more segmented filamentous bacteria, which were found also in a mice model of inflammatory bowel diseases. Mice with low levels of IL-33 are dysbiotic, with a higher concentration of pro-inflammatory bacteria [70]. According these reports, IL-33 could play as a modulator of intestinal inflammation through the interaction with the gut microbiome [71].

**Figure 1:**
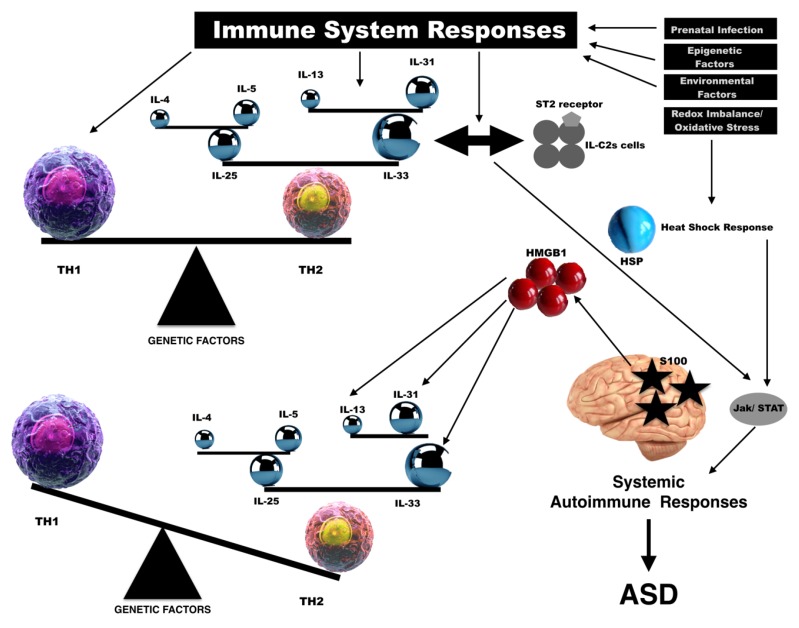
Alarmins involvement in the immune system balance in relation to autism spectrum disorders (ASDs). IL: interleukin; TH: T helper 1/2 immune response; ST2: suppressor of tumorigenicity 2; IL-C: Innate lymphoid cells; HSP: Heat shock protein; HMGB1: High mobility group box-1; Jak/STAT: Janus kinase/Signal Transducer and Activator of Transcription protein.

Although data on IL-33 are still controversial, for sure CD3+ T cells produce cytokines of Th2 type and play a main role in the activation of effector cells during the inflammatory response [72]. IL-25 and IL-33 polarize dendritic cells and promote Th2 responses. These mediators in turn act on dendritic cells causing the activation of other cytokines such as IL-4, IL-5, IL-13 and IL-31 [73,74]. An imbalance of these cytokines, skewed toward Th2, is seen in allergic responses and some systemic autoimmune responses [75,76]. A skewing toward Th1 cytokines was seen in some organ specific immune-dependent disorders such as insulin-dependent diabetes mellitus and multiple sclerosis [77]. Abnormalities in the adaptive responses, effected by cytokines produced by Th cell subsets, was also reported in ASD subjects [78]. These Th1/Th2 responses abnormalities and the intervention of pro-inflammatory cytokines such as IL-33 were reported in condition related to an immune system disorder [3]. On these bases, we speculated an important involvement of some alarmins on the immune system as main etiological actor and regulation in ASDs as reported in Figure 1.

During last years, some studies about vitamin D showed its role in brain development and neuronal functions but also in different psychiatric diseases such as ASD [79]. Furthermore, vitamin D has a neuroprotective role, antioxidant effects, effects on neurotransmission and neuroplasticity, and on cytokines it leads to an anti-inflammatory pattern. In fact, vitamin D deficiency during the gestational period increases the risk of ASD development [80,81,82]. Finally, despite having sometimes a controversial role, vitamin D showed the ability to prevent metabolic and immune-related diseases if taken during pregnancy [83]. This effect might be due to its functions in a correct neurodevelopment [84]. Vitamin D shows its effects by binding to the vitamin D receptor (*VDR*) gene localized in brain tissues and peripheral nerves. In particular, a mutation affecting the *VDR* gene could inhibit the metabolism of vitamin D and cause neuronal development disorders [85]. Recent researches reported that there is a relationship between *VDR* gene polymorphism and ASD [86,87].

## 7. Conclusions

Some alarmins could be suitable as biomarkers of inflammation in ASD. On the other hand, interfering with the immune system by blocking pro-inflammatory mediators could be the key for ameliorating symptoms and behaviours in autistic disorders. The targets could be the proteins themselves or the respective receptors. Further studies, evaluating the efficacy of such molecules on animal studies should be performed, since some monoclonal drugs are already in an experimental phase for other immune related disorders. Individuating, preventing or even treating ASD during the pre-natal period have to be a must and increasing alarmins studies could be an important step.

## Figures and Tables

**Table 1:** List of the original articles concerning alarmins in human autism spectrum disorder (ASD) patients.

Author	Year	No. of Patients	Age	Tissue	Alarmin	Correlation
Barbosa et al. [21]	2015	30	Adults	Blood	IL-33	No differences
Tsilioni et al. [22]	2015	40	Children	Blood	IL-33	No differences
Saresella et al. [23]	2016	25	Children	Blood	IL-33	−
Emanuele et al. [24]	2010	22	Adults	Blood	HMGB1	+
Russo [25]	2013	38	Children	Blood	HMGB1	+
Russo [26]	2014	33	Children	Blood	HMGB1	+
Babinská et al [4]	2014	31	Children/Adults	Plasma	HMGB1	+
Vojdani et al. [27]	2004	50	Children	Blood	Hsp-60	+
El-Ansary et al. [28]	2012	20	Adults	Blood	Hsp-70	+
Ahlsen et al [29]	1993	47	Children	Cerebrospinal fluid	Glial S100	No differences
Boso et al. [30]	2006	18	Adults	Blood	S100A9	+
Coffin et al. [31]	2009	1	Child	Lipoblastoma	S100	+
Al-Ayadhi et al. [32]	2012	64	Children	Blood	S100B	+
Esnafoglu et al. [33]	2017	35	Children	Blood	S100B	No differences
Guloksuz et al. [34]	2017	40	Children	Blood	S100B	+

IL-33: interleukin-33; HMGB1: High mobility group box-1; Hsp-60: Heat shock protein-60.
